# Acquisition of natural humoral immunity to *P. falciparum* in early life in Benin: impact of clinical, environmental and host factors

**DOI:** 10.1038/srep33961

**Published:** 2016-09-27

**Authors:** Célia Dechavanne, Ibrahim Sadissou, Aziz Bouraima, Claude Ahouangninou, Roukiyath Amoussa, Jacqueline Milet, Kabirou Moutairou, Achille Massougbodji, Michael Theisen, Edmond J. Remarque, David Courtin, Gregory Nuel, Florence Migot-Nabias, André Garcia

**Affiliations:** 1 Institut de Recherche pour le Développement, UMR 216 Mère et enfant face aux infections tropicales, Paris, France; 2 COMUE Sorbonne Paris Cité, Université Paris Descartes, Faculté des Sciences Pharmaceutiques et Biologiques, Paris, France; 3 Centre d’Etude et de Recherche sur le Paludisme Associé à la Grossesse et l’Enfance (CERPAGE); Faculté des Sciences de la Santé, Cotonou, Bénin; 4 Laboratoire de Biologie et Physiologie Cellulaires, Faculté des Sciences et Techniques (FAST), Université d’Abomey-Calavi, Cotonou, Bénin; 5 Department for Congenital disorders, Statens Serum Institut, Copenhagen, Denmark; 6 Centre for Medical Parasitology at Department of International Health, Immunology and Microbiology, University of Copenhagen and Department of Infectious Diseases, Copenhagen University Hospital, Rigshospitalet, Copenhagen, Denmark; 7 Biomedical Primate Research Centre, Department of Parasitology, Rijswijk, The Netherlands; 8 Laboratoire de Mathématiques Appliquées (MAP5) UMR CNRS 8145 Université Paris Descartes, Paris, France

## Abstract

To our knowledge, effects of age, placental malaria infection, infections during follow-up, nutritional habits, sickle-cell trait and individual exposure to *Anopheles* bites were never explored together in a study focusing on the acquisition of malaria antibody responses among infants living in endemic areas.Five hundred and sixty-seven Beninese infants were weekly followed-up from birth to 18 months of age. Immunoglobulin G (IgG), IgG1 and IgG3 specific for 5 malaria antigens were measured every 3 months. A linear mixed model was used to analyze the effect of each variable on the acquisition of antimalarial antibodies in 6-to18-month old infants in univariate and multivariate analyses. Placental malaria, nutrition intakes and sickle-cell trait did not influence the infant antibody levels to *P. falciparum* antigens. In contrary, age, malaria antibody levels at birth, previous and present malaria infections as well as exposure to *Anopheles* bites were significantly associated with the natural acquisition of malaria antibodies in 6-to18-month old Beninese infants. This study highlighted inescapable factors to consider simultaneously in an immuno-epidemiological study or a vaccine trial in early life.

Naturally-acquired immunity to malaria develops slowly following repeated exposures to *Plasmodium falciparum*. Parasite, host, environmental and behavioral factors are essential to be examined for a better understanding of the acquisition of clinical immunity and thus clinical outcomes of malaria infections. To our knowledge, this was not exhaustively investigated in previous studies. Indeed, Dobaño *et al*. evaluated the age dependence of naturally acquired antibodies to Merozoite Surface Protein (MSP) 1-19, Apical Membrane Antigen 1 (AMA1), and Erythrocyte Binding Antigen 175 only on the basis of past and present parasite exposure, occurrence of previous episodes and neighborhood of residence^1^. Moreover, Sarr *et al*. showed that malaria specific antibody responses increased with age in children under 5 years old^2^. Baird *et al*. clearly showed that host age, independent of cumulative exposure, represents a determinant key of the quantitative and qualitative immunoglobulin G (IgG) response to *P. falciparum*, certainly through an immune system maturation process^3^. In fact, the role of age in this process is complex and cannot be only related to protection increasing with age as a cumulative product of exposure to antigen^4^.

Environmental factors also influence malaria specific antibody levels. Sarr *et al*., concluded that malaria antibody responses differed according to the areas where distinct *Anopheles* species evolve^2^. Drakeley *et al*. have observed significant associations between parasite prevalence, altitude, as well as recent rainfalls and the prevalence of antibodies specific for MSP1-19 in individuals between 3 to 45 years old^5^. Khaireh *et al*. showed a lower risk to be seropositive to *P. falciparum* for people living at more than 1.5 km of lakes and rivers^6^. Brought together, these examples illustrate the necessity to circumscribe the local environmental characteristics that may influence the acquisition of antimalarial antibodies.

Other factors may be incriminated such as malnutrition. Indeed, low levels of IgG directed to SPf66 and the Ring-infected Erythrocyte Surface Antigen have been shown in wasted children^7^,^8^.

The genotype AS of hemoglobin (Hb) is the main host genetic factor that confers a resistance to severe malaria^9^. Williams *et al*. showed that the HbAS protection occurred during the first 10 years of life. The authors proposed that this innate protection would allow the host to be exposed safely to malaria antigens capable of inducing malaria specific immunity^10^.

Finally, placental malaria could influence the development of immunity in newborns since studies suggested that offspring of women with placental malaria are more susceptible to malaria during infancy^11^,^12^. Moreover, Bonner *et al*. showed that the development of malaria specific antibody responses was impaired during the first year of an infant born to a mother with placental malaria at delivery^13^. Dent *et al*., showed that infants born to mothers with pregnancy-associated malaria without MSP1-driven cord blood lymphocyte responses had a lower functional activity of anti-MSP1 antibodies from 18 to 30 months in comparison to the other infants^14^. However, these results are inconsistent in the literature. Malhotra *et al*, did not found any difference in anti-MSP1 and anti-AMA1 IgG levels between infants born to mothers with or without placental malaria^11^.

The present work is based on an epidemiological study already described^12^ in which all these concurrent determinants were collected in order to be explored as a whole in relation to malaria immunity. It focused on the IgG, IgG1 and IgG3 – that seem to be main actors in malaria antibody responses^15^ – specific for 5 malaria antigens selected i) for their abundance on the merozoite surface and thus their accessibility to host antibodies, and ii) because they are promising vaccine candidates that could induce protective immune responses^16^,^17^. The aims were i) to explore the development of IgG, IgG1 and IgG3 responses to *P. falciparum* blood-stage antigens from birth to 18 months and ii) to understand what are the key factors that could influence the emergence of antibody responses in infants.

## Results

### Descriptive results

**Figure 1:**
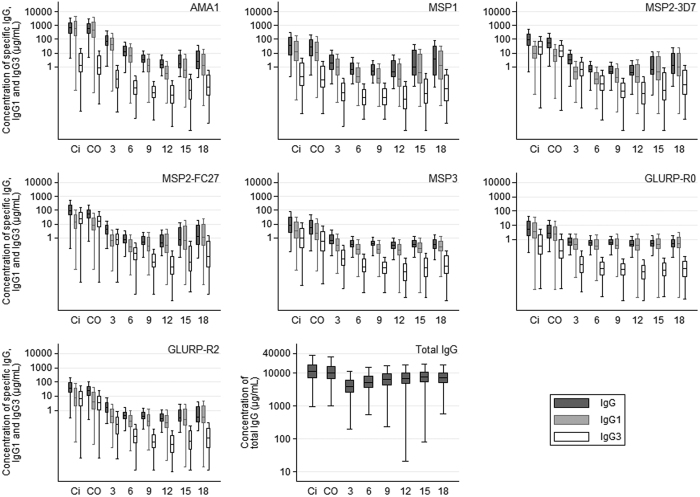
Total IgG and IgG, IgG1 and IgG3 concentrations specific to blood stage antigens in mothers and infants up to 18 months of age Antibody levels determined in: Ci: peripheral maternal blood; CO: cord blood; 3, 6, 9, 12, 15 and 18 months of infants’ age. The full line in the box represents median values. Also shown are 10, 25, 75 and 90% percentiles of the IgG, IgG1 and IgG3 concentrations specific for each malaria antigen. For each time point, the group size is: Ci: n = 525, CO: n = 525, 3: n = 374, 6: n = 384, 9: n = 409, 12: n = 418, 15: n = 431, 18: n = 442.

**Figure 2:**
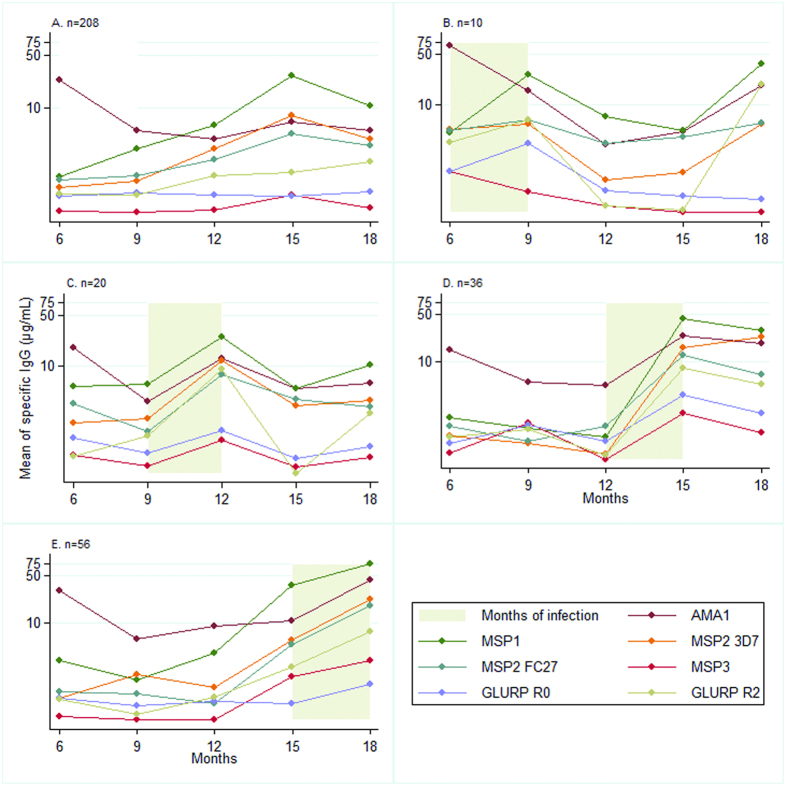
Acquisition of IgG to malaria blood stage antigens for infant with one malaria infection during the follow-up: effect of age (**A**): infants with no malaria infection. Each B-to-E panel concerns distinct children selected on the basis of their first (and only one) malaria infection that occurred either between (**B**) 6–9 months of age or (**C**) 9–12 months of age or (**D**) 12–15 months of age or (**E**) 15–18 months of age; n: number of infants in each group.

**Figure 3:**
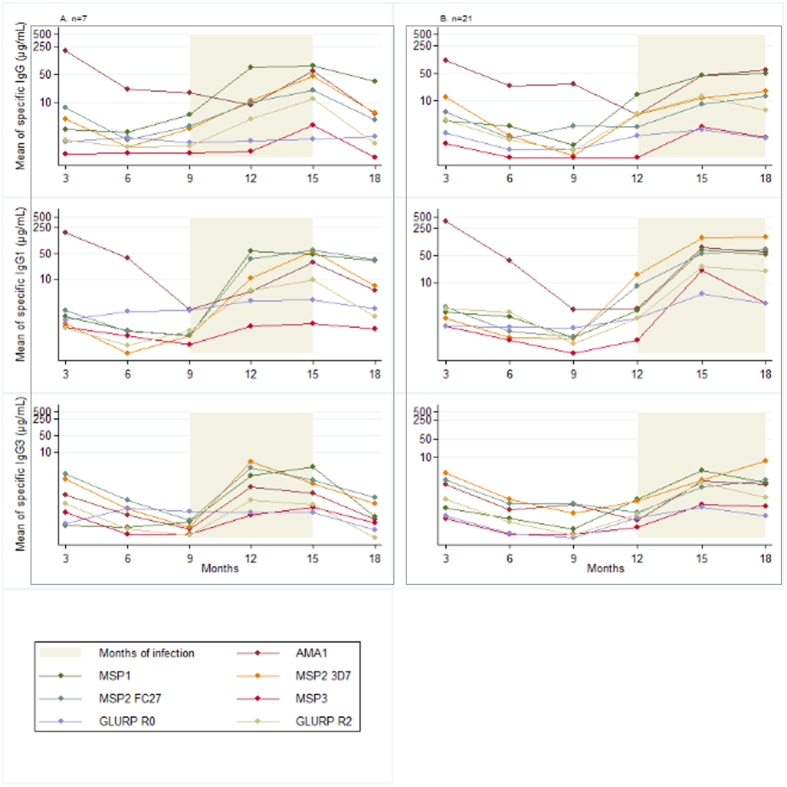
Effect of two successive 3-month periods with malaria infections on the acquisition of IgG, IgG1 and IgG3 to malaria blood stage antigens The panel (**A**) represents the mean concentration of IgG, IgG1 and IgG3 specific for malaria antigens in the 7 infants with a first malaria infection between 9–12 months and a second infection between 12–15 months. The panel (**B**) represents the mean concentration of IgG, IgG1 and IgG3 specific for malaria antigens in the 21 infants with a first malaria infection between 12–15 months and a second infection between 15–18 months; n: number of infants in each group.

The results presented in the Figs 1, 2 and 3 showed the raw data of IgG, IgG1 and IgG3 acquired in concentration (μg/mL) without statistical test associated.

From birth to 6 months of age, maternal IgG, IgG1 and IgG3 are decreasing in infant blood and after 6 months of age the infant antibodies are produced. This production is increasing with age as we can visualize that at 6 months the level of specific IgG, IgG1 or IgG3 and total IgG are lower than at 18 months of age. For AMA1, the concentration of specific IgG/IgG1/IgG3 is stronger than for all the other antigens. The same effect is still observed but instead to see a decrease/increase at 6 months of age, the phenomenon is observed at 12 months of age (raw data in Fig. 1). For all antigens, including MSP2 from the age of 6 months, IgG1 represented the major part of the IgG response. Concerning MSP2 isoforms, IgG3 levels in mother, cord and 3-month blood were i) higher or equal to IgG1 levels and ii) higher than IgG3 levels directed to the other antigens.

Infants with no detected malaria infection during the follow-up were selected in the Fig. 2A. Antibody levels are never null (consistent with undetected infections) but still lower than in infants with at least one infection. In graphs B to E, infants infected only once during the follow-up were selected. Antibody responses following a malaria infection were represented by a positive slope. Antibody levels increased with age and this effect was more marked for infections occurring after 1 year of age.

Figure 3 illustrates the boosting effect on the IgG, IgG1 and IgG3 levels of two consecutive infections during a 6-month observation window. For most antigens, the second infection seems associated with an increasing specific IgG response whereas this trend is less clear for IgG1 and IgG3. The multivariate analysis will examine these results in more depth.

### Univariate analyses

The nutritional status was not associated with antibody levels (*P* > 0.20). Although the sickle-cell trait was negatively associated with the acquisition of IgG3 to MSP2-FC27, this effect was not significant (*P* = 0.26). IgG levels to *P. falciparum* in infants aged more than 6 months were positively associated with maternal (all *P* < 0.001 except IgG3 to MSP2-FC27 (*P* = 0.021) and to MSP3 (*P* = 0.004)), cord (all *P* < 0.001 except IgG3 to GLURP-R2 (*P* = 0.017)) and 3-month-infant (all *P* < 0.001) antibody levels with the strongest effect observed in cord blood. Placental malaria infection was not associated with specific IgG levels.

### Multivariate final model

**Figure 4:**
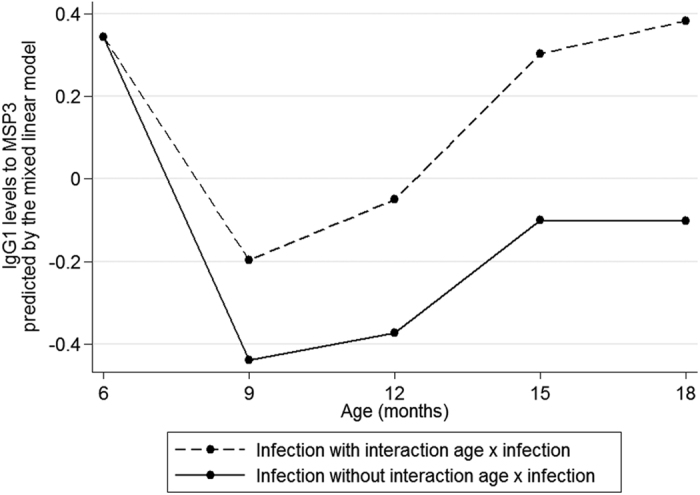
Level of IgG1 specific to MSP3 predicted by the mixed linear model IgG1 specific to MSP3 levels are the residuals of adjustments (cf Materials and Methods -Outcome variable). The effect of an infection at each 3-month period is estimated in presence or not of an interaction between age and infection. The best fitted model includes an interaction between age and infection: the difference between the 2 curves increases with age illustrating that more an infant is old and stronger is his response to a first malaria infection.

Table 1 shows the final model for each antibody response in infants aged more than six months. Overall, age, malaria specific antibody levels in cord blood and at 3 months, an infection having occurred either at the 3-month period or at the 3-to-6-month period before antibody measurement and environmental factors were significantly associated with the acquisition of antigen-specific IgG (data not shown), IgG1 and IgG3 in 6-to-18-month old infants. Age has a polynomial effect and, after a decrease from 6 to 9 or 12 months (depending on antibody), all the levels increased regularly until the age of 18 months (data not shown) as illustrated by the raw data in Fig. 1. Infections at 3–6 months of age were not significant, probably due to the immaturity of the newborn immune system and to the amount of maternal antibodies that could hide the slow acquisition. Contrarily, infections occurring either at the 3-month period or at the 3-to-6-month period before antibody measurement were positively significantly associated with the levels of antibodies. However, the effects of age and of infection on the evolution of antibody levels are complex, and a strong interaction (p < 10^−3^ for each antibody response) was detected between age and infections. This result is consistent with the fact that an infection occurring later in the follow-up had a stronger effect on the antibody response than an infection occurring early after birth when the immune system can be considered as less mature (Fig. 4). This effect of age has been already shown by Baird *et al*., who hypothesized that heavy exposure to infection among children, whether recent or lifelong, does not induce adult-like protection due to the maturation of adult immune system compared with infants^3^. However, we also showed that infection having occurred at the 3-to-6-month period before antibody measurement significantly increased the antibody response showing that the repetitions of infections and, indirectly the delay from the last infection, is an important factor in the acquisition of immunity. The environmental variable was very often significantly associated with high antibody levels (Table 1). Placental malaria and sickle-cell trait were not significant in the multivariate analyses.

## Discussion

The purposes of this work were i) to explore the acquisition of IgG, IgG1 and IgG3 responses to *P. falciparum* blood-stage antigens from birth to 18 months and ii) to highlight the factors that could influence the acquisition of these antibody responses in infant.

Infants with no detected malaria infection presented low but nonzero *P. falciparum* antibody responses allowing 2 complementary interpretations: i) even with a close survey, an infant can develop unperceived infections and ii) malaria specific immune response would subtly develop in infant following submicroscopic infections. The strong effect of the environmental variable corroborates both explanations: *P. falciparum* exposure was related to the specific antibody response in 6–18 months old infants. This variable was inseparable from the presence of anopheles^18^ and furthermore was found strongly associated with the occurrence of the first malaria infection in the same cohort^19^. Pombo *et al*. showed that protection could be induced by sub-clinical infections with very low circulating parasites^20^ allowing to conclude that residing in a malaria endemic area is sufficient to induce a subtle development of anti-plasmodial immune responses. The present results underline the importance to take into account as precisely as possible the exposure to *P. falciparum* during sero-epidemiological studies.

An infection occurring in the 3-to-6-month period before the antibody measurement could be followed or not by an infection in the 3-month period. In case of no infection occurring during the 3-month period before the antibody measurement, there was a positive “residual effect” on the level of total and cytophilic IgG, of the infection having occurred in the last 3-to-6 month period. In case of an additional infection during the 3-month period before the antibody measurement, the malaria specific antibody levels were boosted (2 consecutive infections). This is consistent with the development of an immune memory for infants living in an endemic area. Additionally, the quantification of memory B cells (CD19+ CD21+ CD27+) would represent a substantial information especially as Ngundu *et al*., did not show any correlation between malaria antibody levels and memory B cell frequencies^21^.

In the present study, the 6–18 months infants’ levels of antimalarial antibodies were not related to placental malaria. These results are consistent with those of Malhotra *et al*. who followed-up a birth cohort in Kenya and found no age-linked differences in anti-malaria IgG levels between not exposed, exposed-and-not-sensitized and sensitized groups of children^11^. Contrarily, Bonner *et al*. showed that antimalarial antibody responses decreased during the first year of an infant born to a mother with placental malaria at delivery^13^. However, these authors compared time-pooled measures (0–4 months and 4–12 months) that could amplify the differences between infants born to mothers with and without placental malaria at delivery. In the birth cohort under study, infants born to mothers with placental malaria developed a malaria infection earlier compared to others^12^. According to the present results, the evolution of the infants’ malaria antibody responses seems not to be involved in this phenomenon. Indeed, Malhotra *et al*. also observed that tolerant (exposed-and-not-sensitized) children had impaired functional antibody responses to MSP1-19^14^. An investigation of the impact of antimalarial antibodies on protection to clinical malaria would lead to conclude on the role of these antibodies in infants born to mothers with placental malaria.

Cord blood malaria antibody levels were positively associated with *P. falciparum* IgG, IgG1 and IgG3 levels during the follow-up were not associated with placental malaria. This result means that the infants born with high levels of transplacental transferred maternal antibodies to malaria produced high levels of IgG specific for malaria. If we consider the hypothesis according to which antibodies are markers of exposure, the higher malaria infections would occur in infants the higher the malaria antibody levels would be. It was previously demonstrated in this cohort that infants born to mothers with placental malaria had a higher risk of malaria infection in their 18 first months of age^19^. Antibodies as markers of exposure could thus potentially explain this result. The literature provides contradictory conclusions regarding maternal antimalarial antibodies transferred to the fetus and protection of infant from early malaria infections^22^. Regarding the present study, the number of malaria infections up to the age of 6 months was not associated with the transfer of malaria-specific IgG from the mother to the fetus, leading to the conclusion that malaria-specific IgG represented a marker of maternal exposure rather than a marker of infant protection from malaria^23^.

Nutritional intakes had no effect on malaria specific antibody levels. Genton *et al*. found no association between antibody levels to MSP1 and MSP2 and nutritional status^7^. However, in the present study nutritional intakes were assessed by means of 24-Hour Dietary Recall questionnaire and not by nutrients measurements whereas nutrients are constitutional components of the immune response^24^. Among them, zinc deficiency has been shown to compromise the B lymphocyte development and therefore the antibody production^25^. Further investigations on the nutritional status at the nutrient level and its impact on the acquisition of malaria specific immunity are necessary.

Sickle-cell trait was not significantly associated with antibody evolution in the univariate or multivariate analyses.

In the present work, antibody responses were analyzed independently. The profiles of malaria antibody concentrations in infant are similar over the time as those expressed in arbitrary units or in OD values in other studies^26^,^27^,^28^. Antibody response curves vary depending on merozoite antigens and IgG subclasses - as illustrated by the higher level of IgG3 specific for MSP2 in women (as described elsewhere^29^) - and they result from the sum of individual responses, each being characterized by defined subpopulations of IgG antibody-secreting cells^28^. The next step will consist to take into account the synergy of the antibody responses directed to the five studied antigens, in order to obtain for each infant a fine picture of the acquisition process of malaria humoral immunity^30^.

At last, this study demonstrates the huge importance to give to key factors conditioning the antibody response in early life (age, malaria antibody levels at birth, previous and present malaria infections as well as exposure to *Anopheles* bites). Such factors have to be considered simultaneously in follow-ups of cohorts aimed at elucidating the specific humoral response mainly in the context of vaccine trials.

## Methods

### Study design and sample collection

The study took place in Southern Benin where *P. falciparum* is the commonest species (95%)^31^ and where the entomological inoculation rate is 15.48 infected anopheles/person per year^12^. Between June 2007 and July 2008, 656 newborns were enrolled and 567 infants remained for this study (10 stillbirths, 16 infants deceased before Day 28, 10 infants lost of sight, 25 twin pairs and one set of triplets)^12^.

Briefly, at delivery, (i) a questionnaire collecting information on maternal age, parity, use of Intermittent Preventive Treatment during pregnancy (IPTp) and bed net possession was administered and (ii) placental blood smears were performed and maternal peripheral as well as cord bloods were collected into Vacutainer^®^ EDTA (Ethylene diaminetetraacetic acid) tubes.

Every child was visited weekly. In case of fever (axillary temperature ≥37.5 °C) and/or a reported 48-hours-fever episode, a questionnaire and both a rapid diagnostic test (RDT) and a thick blood smear (TBS) were performed. Symptomatic malaria infection (fever and positive TBS and/or RDT) was treated with artemether and lumefantrine combination therapy as recommended by the National Malaria Control Program. Every month, a systematic TBS to detect asymptomatic malaria infection and a qualitative dietary questionnaire^32^ were performed. Every 3 months (at 3, 6, 9, 12, 15, 18 months of age), infant blood samples were collected (in EDTA). Plasmas were conserved at −80 °C.

### Antibody measurements

#### Antibody measurement by Enzyme-Linked ImmunoSorbent Assay (ELISA)

ELISA was performed as described previously^17^ and recalled in the Supplementary material^33^,^34^,^35^,^36^,^37^,^38^. The Afro Immuno Assay protocols - standard methods developed for evaluating malaria vaccines sponsored by the African Malaria Network Trust (AMANET [www.amanet-trust.org]) - were performed to assess malaria antibody concentrations by ELISA^39^,^40^.

Standard curves were established using human IgG, IgG1 and IgG3 purified proteins (The Binding Site, France) to determine the concentration of specific antibodies. Each point was tested in duplicate.

#### Data Management for ELISA data

The ADAMSEL FLP b039 software (http://www.malariaresearch.eu/content/software) was used to analyze automatically the ELISA optical density (OD) leading to antibody concentrations. Discordant duplicates (with a variation coefficient >15%) were technically repeated.

OD that were below detection threshold or over saturation were referred as “Low” and “High” concentrations (μg/mL) respectively. These thresholds depend on standard concentrations, normalization procedures and are therefore specific to each ELISA plate. These censored values were imputed using a (log) linear regression model taking into account confounding variables. The model was fitted using a stochastic expectation maximization algorithm^41^ already applied to ELISA analyses^42^, and estimated values were attributed to the “Low” and “High” censored values. The quality of imputation, assessed by the R2 between imputed and measured values (both in log-scale), was very good for both low (R2 = 0.9082 for the 5156 concerned measurements) and high (R2 = 0.9498 for the 192 concerned measures). The residuals were classically standardized to obtain a Normal distribution. The R2 between the imputed values and adjusted residuals (both in log-scale) was 0.9906.

### Outcome variable: adjusted antibody level

The outcome variable was time-dependent (at 6, 9, 12, 15 and 18 months). Antibody levels were firstly adjusted on the nuisance variables using a linear regression for: health center, prematurity, parity, use of IPTp, bed net possession and maternal age in order to focus on the effects of variables of interest (detailed in “Independent variables” section). The residuals of these adjustments - referred as “adjusted antibody level” - were then used as outcome variable.

### Independent variables

#### Maternal, cord and 3-month infant blood samples

Birth and 3-month infant samples are strongly represented by maternal antibodies that could interfere with the evaluation of acquired IgG of neonates^43^. Antibody measures in these samples were thus used as quantitative variables in the analyses.

#### Infections

Placental malaria infection was defined by the presence of *P. falciparum* in thick placental smears.

The number of malaria attacks between two successive antibody measurements was used either as a quantitative or a qualitative variable. Malaria infections could be feverish or not.

The effect of an infection on the antibody levels was tested by two ways: a prospective effect and a boosting effect of a second infection. To evaluate the prospective effect, infections occurring in the 3-month period before the antibody measurements were considered to be associated with antibody levels. To evaluate the boosting of the antibody response in children being infected in the 3-month period before the antibody measurement, we considered whether they were also infected in the 3-to-6 month period (2 consecutive infections: one occurring in the 3-to-6 month period and one in the 3-month period before the antibody measurements).

#### Infant’s feeding

Individual 24-Hour Dietary Recall questionnaires - based on child feeding practices^32^ - were administered monthly from birth. A time-dependent quantitative variable accounting for the past 3-month feeding practices was created^12^.

#### Sickle-cell trait

Genomic DNA was extracted from buffy coat using QIAamp DNA blood midi kit (Qiagen, France). The amplified beta globin DNA was then cleaved by the *DdeI* enzyme into 3 fragments when normal and into 2 fragments when mutated^44^.

#### Environmental factors

The environmental risk of exposure to malaria was modeled for each child based on climatic, entomological, and characteristics of the children immediate surroundings^18^. Using this model an individual time-dependent risk of exposure was attributed to each child, depending on his birthday and on his house’s location in the area.

### Statistical analyses

During the study period, antibodies levels were determined quarterly, and the child’s measurements cannot be considered independent. Therefore, child’s antibody levels presented a two-level hierarchy: antibody measurements (level 1) were clustered within children (level 2)^45^. A linear mixed model with assumed independent random-coefficients was used for both univariate and multivariate analyses. Hierarchical mixed models are well suited to this type of data, allowing analyzing the time dependent evolution of antibodies, and were used in this study^45^ for both univariate and multivariate analyses. Variance components and fixed effects parameters were estimated using the restricted and the not restricted maximum likelihood methods, respectively^45^.

The effect of age on the acquisition of natural antibodies was tested using polynomial models. The best fitted model (Bayesian Information Criterion) was used for further analyses.

Multivariate analysis was performed including the variables with *P* ≤ 0.20 in the univariate step. Placental malaria was systematically included in multivariate analyses. An interaction between age and infection was tested in the model. All statistical analyses were performed using Stata, version 11.0 (StatCorp LP, TX, USA). Statistical significance was set at *P* < 0.05.

### Ethics

The study protocol was approved by the University of Abomey-Calavi’s institutional review board and the IRD’s Consultative Ethics Committee. All women in this study signed an informed consent before enrollment (which also included their children) with the possibility to withdraw at any time. All the methods were carried out in accordance with the approved guidelines.

## Additional Information

**How to cite this article**: Dechavanne, C. *et al*. Acquisition of natural humoral immunity to *P. falciparum* in early life in Benin: impact of clinical, environmental and host factors. *Sci. Rep*. **6**, 33961; doi: 10.1038/srep33961 (2016).

## Supplementary Material

Supplementary Information

## Figures and Tables

**Table 1:** Multivariate analysis: final model indicating factors associated with the acquisition of malaria specific cytophilic IgG in infants from 6 to 18 months of age.

Variables	AMA1		MSP1		MSP2-3D7		MSP2-FC27		MSP3		GLURP-R0		GLURP-R2	
	IgG1	IgG3	IgG1	IgG3	IgG1	IgG3	IgG1	IgG3	IgG1	IgG3	IgG1	IgG3	IgG1	IgG3
Constant	3.819	−1.180	−1.487	−4.182	3.784	−0.961	−2.805	−0.322	−2.350	−2.834	−2.171	3.603	−1.685	−2.410
Age	−1.018^a^	−0.581	−0.325	−0.181	0.090	−0.781	0.068	−0.862	−0.078*	−0.419	0.050	−0.224	−0.189	−0.552
Age squared	0.037	0.023	0.015	0.008	NS	0.031	NS	0.033	0.005**	0.017	NS	0.009	0.010	0.022
Antibody levels in cord blood	0.131	0.097**	0.132	0.060*	0.148	0.209	0.072	0.114**	0.085**	NS	0.073**	0.126*	NS	NS
Antibody levels at 3 months	0.063**	0.135	0.113	0.206	0.129	0.173	0.147	0.140	0.183	0.206	0.147	0.196	0.164	0.169
Infection at 3–6 months of age	NS	NS	NS	−0.345**	0.244*	NS	NS	NS	NS	NS	NS	−0.406	NS	NS
Infection from 6 months of age:														
in the 3-month period preceding antibody measurement	1.335	1.445	2.210	2.222	1.716	1.826	1.696	1.778	0.633	0.945	0.588	0.834	1.436	1.362
in the 3-to-6-month period preceding antibody measurement	1.443	1.114	1.909	1.277	1.519	1.343	1,494	1.439	0,380	0.51	0.221*	0.240**	0.697	0.499
Environmental variable	0.032	0.024**	0,027*	0,040**	0.039	0.029	NS	0,038**	NS	NS	0,039	0,019*	NS	0,024*

a: The numbers of the entire Table 1 are coefficient of mixed linear regression: a positive (negative) coefficient indicates a positive (negative) effect of the variable on antibody levels from 6 until 18 months of life. All associations are significant at a *P* value of ≤0.001 unless otherwise indicated: NS non-significant, *P < 0.05, **P ≤ 0.01, with quarterly febrile infection cases, with quarterly non febrile infection cases. Malaria infections in the previous 3-month period are defined as the presence of at least one malaria attack between two successive antibody measurements and could be feverish or not. The infections in the previous 3-to-6-month period allowed the creation a variable for testing the effect of 2 consecutive infections (separated by at least 3 months) on malaria specific antibody responses. Age is in months; Infections are coded as: at least one infection = 1, no infection = 0; antibody levels are represented by quantitative residual values; the environmental variable is a quantitative index related to the malaria exposure of the infant in the area (described elsewhere^18^).
