# Supplementary material for: Acquisition of natural humoral immunity to P. falciparum in early life in Benin: impact of clinical, environmental and host factors
Source: Sci Rep. 2016 Sep 27;6:33961. doi: 10.1038/srep33961 (PMC5037375; doi:10.1038/srep33961)
Supplement: Supplementary Information [file srep33961-s1.pdf]

Acquisition of natural humoral immunity to P. falciparum in early life in Benin: impact of clinical, environmental and host factors

Célia Dechavanne*,1,2, Ibrahim Sadissou3, Aziz Bouraima3, Claude Ahouangninou3, Roukiyath Amoussa3, Jacqueline Milet1,2, Kabirou Moutairou4, Achille Massougbodji4, Michael Theisen5,6, Edmond J. Remarque7, David Courtin1,2, Gregory Nuel8, Florence Migot-Nabias1,2, André Garcia1,2

Supplementary Material 1: Recombinant protein information and ELISA dilutions

Recombinant protein information						ELISA dilutions		
Recombinant antigen	Strain	Amino acids positions	System of expression	Laboratory source	References	IgG classes	Dilution for maternal, cord and 3-month infant blood samples	Dilution for 6-, 9-, 12-, 15-, 18-month infant blood samples
AMA1	FVO	25-545	Pichia pastoris	The Biomedical Primate Research Centre (Rijswijk, The Netherlands)	[1]	Total IgG	1:2000	1:500
						IgG1	1:1000	1:50
						IgG3	1:100	1:50
MSP1-19	Uganda-Palo-Alto	1-43 and 1615-1723	Baculovirus/insect	Pasteur Institute (Paris, France)	[2]	Total IgG	1:200	1:100
						IgG1	1:200	1:50
						IgG3	1:500	1:50
MSP2-3D7	F32	whole protein without the secretion signal and GPI anchor	E. coli	La Trobe University (Melbourne, Australia)	[3,4]	Total IgG	1:200	1:100
						IgG1	1:200	1:50
						IgG3	1:500	1:50
MSP2-FC27	F32	whole protein without the secretion signal and GPI anchor	E. coli	La Trobe University (Melbourne, Australia)	[3,4]	Total IgG	1:200	1:100
						IgG1	1:200	1:50
						IgG3	1:500	1:50
MSP3	F32	212-380	E. Coli	Statens Serum Institute (Copenhagen, Denmark)	[5,6]	Total IgG	1:200	1:100
						IgG1	1:200	1:50
						IgG3	1:100	1:50
GLURP-R0	F32	25-514	E. Coli	Statens Serum Institute (Copenhagen, Denmark)	[5,6]	Total IgG	1:200	1:100
						IgG1	1:200	1:50
						IgG3	1:100	1:50
GLURP-R2	F32	706-1178	E. Coli	Statens Serum Institute (Copenhagen, Denmark)	[5,6]	Total IgG	1:200	1:100
						IgG1	1:200	1:50
						IgG3	1:500	1:50

Legend: An anti-human IgG1 (NL16 clone) diluted 1:2000 and an anti-human IgG3 (ZG4 clone) diluted 1:5000 (Skybio, France) were used as well as a peroxidase-conjugated goat anti-mouse IgG diluted 1:3000 (Invitrogen, France) for revealing the reaction.
